# Supplementary material for: Racial disparities in urine drug screening among seizure patients in the emergency department
Source: PLoS One. 2025 Sep 10;20(9):e0331908. doi: 10.1371/journal.pone.0331908 (PMC12422493; doi:10.1371/journal.pone.0331908)
Supplement: Attachment — Submitted filename: Response letter.docx [file pone.0331908.s002.docx]

June 21, 2025

We thank the reviewer for the thoughtful feedback on our manuscript**.** We have carefully considered all comments and have made revisions to improve the quality and clarity of this work. We have updated our manuscript and have included new references that strengthen our results. Below is a detailed response to each of the reviewer’s comments with responses in bold:

Reviewer #1: Reviewer comment and suggestions

Title
• The title is clear and informative, accurately reflecting the study's focus on racial disparities in urine drug screening (UDS) among patients presenting with suspected seizures. **We appreciate your input about the title.**

Abstract:
• An abstract summarizing the background, methods, results, and conclusion will enhance accessibility and provide quick insight into the study’s contributions. An abstract following PLOS guidelines should also include keywords.
**We concur with this statement. Our abstract summarizes the background, methods, results, and conclusion. The most relevant data have been highlighted in this section. Five keywords are included in accordance with PLOS guidelines.**

Introduction:
• The introduction could benefit from a more thorough literature review. Acknowledging existing studies on UDS disparities would strengthen the foundation for this research. Consider including the rationale for focusing specifically on UDS and the clinical implications of equitable access to this testing.
**This comment is very insightful, and the introduction has been updated accordingly. As this manuscript was initially written in 2022, newer studies were not included in the original draft. We have now incorporated updated and existing references (1–7), which strengthen the rationale and context of our work: although literature evaluating racial disparities in seizure care is rising, data addressing racial disparities in ED drug testing in seizure patients is still sparse. Thus, we were interested on focusing on UDS testing rates in patients evaluated for seizures at the emergency department.**

Methods:
• A clear description of sample selection criteria is provided, but details about exclusion criteria or specific demographic data (e.g., age ranges, comorbid conditions) would enhance reproducibility.
**We appreciate these comments. In the Methods section, we have clarified that the inclusion criteria considered patients aged 18 years and older, thereby excluding pediatric populations. Additionally, ICD-10 codes for seizures have been added. Patients were excluded if UDS data were unavailable or incomplete. We did not stratify by comorbid conditions; therefore, this information was not included.**

• Information regarding the statistical analysis might be more detailed. Indicating confidence intervals and effect sizes for the Chi-square results would provide a clearer picture of the clinical significance of the findings.
**This information has now been added to the Methods section:
*Differences in UDS regarding positivity rates and repeated UDS frequency were evaluated using the Chi-square test for two categorical variables. A p-value of ≤ 0.05 was considered statistically significant. To enhance clinical interpretability, 95% confidence intervals were calculated for proportions, and effect sizes were considered where relevant.***

Results:
• The presentation of data is straightforward, with clear comparisons drawn between racial groups. However, including visual aids such as tables or graphs to present results (e.g., rates of UDS testing and positivity by race) could improve clarity.
**We concur with this suggestion. Accordingly, we have included four figures and two tables to clearly present our results.**

• The narrative mentions UDS substance positivity but does not elaborate on the potential clinical implications of these findings, such as differences in management or treatment outcomes based on detected substances.
**Thank you for your feedback. The primary aim of our study was to examine racial disparities in the utilization of UDS testing among patients presenting with suspected seizures in the ED. While we did report positivity rates, these were secondary outcomes intended to provide context rather than serve as the focus of our analysis. We chose not to elaborate on clinical management or treatment implications based on detected substances, as this falls outside the scope of our current objectives. Our intention was to highlight how differential use of UDS may reflect or contribute to broader inequities in seizure evaluation.**

Discussion:
• The discussion provides a solid interpretation of findings but could incorporate broader implications of racial disparities in medical testing and treatment. Consider exploring the potential reasons for observed disparities, including systemic biases or differences in healthcare access. **Thank you for this insightful comment. We appreciate the suggestion to broaden the discussion. In response, we have revised the discussion section to incorporate a more in-depth exploration of the potential reasons underlying the observed racial disparities, including considerations of systemic bias and inequities in healthcare access. We have also added new references to support these additions and to provide context for the broader implications of our findings.**

• Addressing the limitations of the study, such as potential biases in retrospective data analysis or unmeasured confounders would enhance the transparency of the research.
**To address this comment, we have added the following statement under the limitations section: *This study is susceptible to unmeasured confounders and selection biases that may have influenced both the exposure and outcome, which we could not fully control due to limitations in the available data.***

• The conclusion reiterates the main findings but could benefit from a call to action, such as recommendations for addressing these disparities in clinical practice or suggestions for future research. **We have revised the conclusion to include a call to action, encouraging further research to address racial disparities in seizure evaluation and UDS utilization in the ED.**

References:
• Ensure that all references are current and relevant, following PLOS guidelines for citation style. A comprehensive reference list will enhance the study's credibility. **References have been updated and formatted according to PLOS guidelines.**

General Formatting:
• Ensure that the manuscript follows PLOS ONE formatting guidelines, including specific font sizes, headings, and citation styles. This attention to detail is essential for submission readiness.
**The manuscript has been revised to comply with all PLOS ONE formatting requirements, including font sizes, headings, and citation style.**

Ethical Considerations:
• Consider including a statement confirming institutional review board approval and patient consent where applicable.
**This statement is included in the Methods section:
*The local institutional review board (IRB) approved this study on October 20, 2020 (IRB STUDY00146470). Since our group utilized deidentified data, this study was deemed exempt, and the IRB waived the requirement for informed consent. The data were accessed for research purposes from October 20, 2020, to June 30, 2021.***

In summary, this study addresses an important topic concerning racial disparities in UDS among patients with suspected seizures. Improvements in clarity and depth of analysis, alongside adherence to PLOS guidelines, will strengthen the manuscript's overall impact and quality.
**We fully agree with this summary. In response, we have updated the manuscript to include new references, improve clarity and writing style, and enhance the overall readiness of the paper for submission. We are excited to share this revised version and believe it is significantly strengthened based on your valuable feedback.**

**Journal Requirements:**

When submitting your revision, we need you to address these additional requirements.

1. Please ensure that your manuscript meets PLOS ONE's style requirements, including those for file naming. **The manuscript has been updated to meet PLOS ONE’s style requirements.**

2. We note that your Data Availability Statement is currently as follows: [All relevant data are within the manuscript and its Supporting Information files.]

Please confirm at this time whether or not your submission contains all raw data required to replicate the results of your study. Authors must share the “minimal data set” for their submission. PLOS defines the minimal data set to consist of the data required to replicate all study findings reported in the article, as well as related metadata and methods (https://journals.plos.org/plosone/s/data-availability#loc-minimal-data-set-definition).

For example, authors should submit the following data:

- The values behind the means, standard deviations and other measures reported;
- The values used to build graphs;
- The points extracted from images for analysis.

Authors do not need to submit their entire data set if only a portion of the data was used in the reported study.

If your submission does not contain these data, please either upload them as Supporting Information files or deposit them to a stable, public repository and provide us with the relevant URLs, DOIs, or accession numbers. For a list of recommended repositories, please see <https://journals.plos.org/plosone/s/recommended-repositories>.

If there are ethical or legal restrictions on sharing a de-identified data set, please explain them in detail (e.g., data contain potentially sensitive information, data are owned by a third-party organization, etc.) and who has imposed them (e.g., an ethics committee). Please also provide contact information for a data access committee, ethics committee, or other institutional body to which data requests may be sent. If data are owned by a third party, please indicate how others may request data access.

**We include this data as supporting information file.**

3. Please upload a new copy of Figure 3 as the detail is not clear. Please follow the link for more information: **We have updated figure 3 which now corresponds to figure 4.**

**Best,**

**Gloria Ortiz Guerrero, MD**
