# Supplementary material for: Racial disparities in urine drug screening among seizure patients in the emergency department
Source: PLoS One. 2025 Sep 10;20(9):e0331908. doi: 10.1371/journal.pone.0331908 (PMC12422493; doi:10.1371/journal.pone.0331908)
Supplement: Attachment — Submitted filename: RESPONSE LETTER.docx [file pone.0331908.s003.docx]

**RESPONSE LETTER**

Dear editor and reviewers:

Thanks for considering our manuscript for publication. Below are the modifications we made to our manuscript as requested:

1. We added the financial disclosures to the cover letter as requested.
2. We expanded the study limitations to include the suggestion provided by Dr. Abdallah. These changes are highlighted in tracked changes on page 12, between line 239 and 240.
3. We used Preflight Analysis and Conversion Engine (PACE) to ensure figures meet PLOS requirements.

Best,

Gloria Ortiz Guerrero, MD
